# Genetic and structural identification of an O-acyltransferase gene (*oacC*) responsible for the 3/4-O-acetylation on rhamnose III in *Shigella flexneri* serotype 6

**DOI:** 10.1186/s12866-014-0266-7

**Published:** 2014-10-21

**Authors:** Yuriy A Knirel, Jianping Wang, Xia Luo, Sofya N Senchenkova, Ruiting Lan, Anna M Shpirt, Pengcheng Du, Alexander S Shashkov, Nan Zhang, Jianguo Xu, Qiangzheng Sun

**Affiliations:** N.D. Zelinsky Institute of Organic Chemistry, Russian Academy of Sciences, Moscow, Russian Federation; State Key Laboratory for Infectious Disease Prevention and Control, Collaborative Innovation Center for Diagnosis and Treatment of Infectious Diseases, National Institute for Communicable Disease Control and Prevention, China CDC, P.O. Box 5, Changping, Beijing, China; School of Biotechnology and Biomolecular Sciences, University of New South Wales, Sydney, NSW 2052 Australia

**Keywords:** *Shigella flexneri*, 3/4-O-acetylation, Acyltransferase, *oacC*, O-antigen, Anti-O-factor 9 serum

## Abstract

**Background:**

O-antigen (O-polysaccharide) of the lipopolysaccharide is a highly variable cell component of the outer membrane in *Shigella flexneri*. It defines the serospecificity and plays an important role in the pathogenesis of shigellosis. There are two distinct O-antigen forms for the 19 serotypes of *S. flexneri*: one for serotypes 1–5, X, Y, 7 (and their subtypes), and the other for serotype 6. Although having different basal O-polysaccharide structures, the two forms share a common disaccharide fragment [→2)-α-l-Rha*p*^III^-(1 → 2)-α-l-Rha*p*^II^]. In serotype 6 and some non-6 serotypes, Rha^III^ is O-acetylated at position either 3 or 4 (3/4-O-acetylation), conferring to the hosts a novel antigenic determinant named O-factor 9. An acyltransferase gene (*oacB*) responsible for this modification has been identified in serotypes 1a, 1b, 2a, 5a, and Y, but not in serotype 6.

**Results:**

Using genetic, serological, and chemical approaches, another acyltransferase gene named *oacC* was demonstrated to be responsible for the 3/4-O-acetylation on Rha^III^ in the O-antigen of *S. flexneri* serotype 6. Inactivation of the *oacC* gene resulted in the loss of the 3/4-O-acetyltion, and the cloned *oacC* gene restored this modification upon transformation. In accordance with the similarity in the acceptor substrate structure and high sequence homology (72% identity) between *oacC* and *oacB*, *oacC* has the interchangeable function with the *oacB* gene in mediation of the 3/4-O-acetylation. The *oacC* gene is located in a prophage on the chromosome and presented in all 77 serotype 6 strains tested.

**Conclusions:**

Identification and functional characterization of the O-acetyltransferase encoding gene, *oacC*, shows that it is involved in O-antigen modification by 3/4-O-acetylation on Rha^III^ specific to serotype 6.

**Electronic supplementary material:**

The online version of this article (doi:10.1186/s12866-014-0266-7) contains supplementary material, which is available to authorized users.

## Background

*Shigella flexneri* is the major pathogen causing bacillary dysentery (shigellosis) in developing countries. It is estimated that there are 125 million shigellosis cases annually in Asia, resulting in 14,000 deaths, the majority of which are children under 5 years old [1]. The O-polysaccharide chain of the lipopolysaccharide (LPS) called O-antigen is an important and highly variable cell component presented on the outer leaflet of the outer membrane. It provides the basis for serotyping of *S. flexneri*, and plays an important role in the pathogenesis of shigellosis [2,3]. The immune response against the O-antigen can induce protection that makes it a promising candidate as a component of shigellosis vaccines [4-7].

Based on the composition and structures of the O-antigens, *S. flexneri* can be divided into two main groups: one for serotypes 1–5, X, Y, and 7, and the other for serotype 6. Members of the former group share a linear O-polysaccharide backbone composed of tetrasaccharide repeats (O-units) of one *N*-acetylglucosamine (GlcNAc) and three l-rhamnose residues (Rha^I^-Rha^III^): →2)-α-l-Rha*p*^III^-(1 → 2)-α-l-Rha*p*^II^-(1 → 3)-α-l-Rha*p*^I^-(1 → 3)-β-d-Glc*p*NAc-(1 → [8,9]. Adding various chemical groups (glucosyl, acetyl or/and phosphoethanolamine) to different sugars of the tetrasaccharide backbone gives rise to diverse O-antigen structures and, correspondingly, to various serotypes [10,11]. Serotype 6 has a different linear O-polysaccharide backbone with a tetrasaccharide repeat containing one residue each of *N*-acetylgalactosamine (GalNAc) and galacturonic acid (GalA) and two rhamnose residues (Rha^II^-Rha^III^): →2)-α-l-Rha*p*^III^-(1 → 2)-α-l-Rha*p*^II^-(1 → 4)-β-d-Gal*p*A-(1 → 3)-β-d-Gal*p*NAc-(1→ [12]. The → 2)-α-l-Rha*p*^III^-(1 → 2)-α-l-Rha*p*^II^ disaccharide fragment is common for the O-polysaccharides of both groups of *S. flexneri*. The observed O-antigen distinction reflects the genetic diversity of *S. flexneri* and different evolutionary origins of serotype 6 compared to other serotypes, which belong to different lineages of *Shigella* clones of *Escherichia coli* [13,14].

Similar to other *Shigella* species, the O-antigen gene cluster involved in the biosynthesis of the *S. flexneri* O-polysaccharide backbone is located between the housekeeping genes *galF* and *gnd* on the chromosome [15]. It contains three main classes of genes: (i) genes of nucleotide sugar biosynthesis pathways; (ii) glycosyltransferase genes; and (iii) O-unit processing genes including those for O-antigen flippase (*wzx*) and O-antigen polymerase (*wzy*). Serotypes 1–5, X, Y, and 7 have similar O-antigen gene clusters whereas in serotype 6 the gene cluster is different, resulting in the two dissimilar O-antigen backbone structures of *S. flexneri* [15]. The factors responsible for modification of the backbone including O-antigen glucosylation (*gtr* cluster), O-acetylation (*oac* and *oacB*), and phosphorylation (*opt*) are carried by prophages, transposon-like structures, or plasmids outside of the O-antigen gene cluster [10,11,16,17].

Modification of the O antigen by O-acetylation has been also found in the serotype 6. It was first reported at position 3 of Rha^III^ [12], and later found that Rha^III^ is partially 3-O-acetylated (major type) and partially 4-O-acetylated (minor type) [18]. The degree of 3/4-O-acetylation varies between strains and is thought to contribute to the serospecificity, which gives rise to a serological distinction between strains with lower (~30% and 15%) and higher (~60% and 30%) degrees of O-acetylation at position 3 and 4 (serotypes 6 and 6a, respectively) [18].

A similar 3/4-O-acetylation on Rha^III^ also occurs in serotypes 1a, 1b, 2a, 5a, and Y of *S. flexneri* [16,18,19], and the O-acyltransferase-encoding gene, named *oacB*, has recently been found to be responsible for this modification in these serotypes [16]. *oacB* is carried by a transposon-like structure located upstream of the *adrA* gene on the chromosome [16]. Further studies have indicated that the 3/4-O-acetylation on Rha^III^ is widespread in serotypes 1a, 1b, 2a, 5a, 6, and Y and confers to the bacterium a novel antigenic determinant provisionally named group O-factor 9 [20]. However, the *oacB* gene cannot be detected from serotype 6 strains carrying 3/4-O-acetylation [16], indicating that another unknown gene is involved in the O-antigen modification in this serotype.

Considering that the O-antigen plays a key role in the serospecificity and virulence of *Shigella*, elucidation of O-antigen modification mechanisms is important for understanding the O-antigen biosynthesis, antigenicity, and pathogenicity of *S. flexneri*, as well as for shigellosis vaccine development. In this study, we identified the acyltransferase gene, named *oacC,* which mediates the 3/4-O-acetylation on Rha^III^ in serotype 6. The *oacC* gene is located in a phage-like structure on the chromosome and has an interchangeable function with the *oacB* gene of serotypes 1a, 1b, 2a, 5a, and Y. However, PCR screening indicated that gene *oacC* is specific to serotype 6, whereas *oacB* is specific to the other serotypes of *S. flexneri.*

## Results and Discussion

### Identification of an O-acyltransferase gene, *oacC*, on the chromosome of *S. flexneri* serotype 6

**Figure 1:**
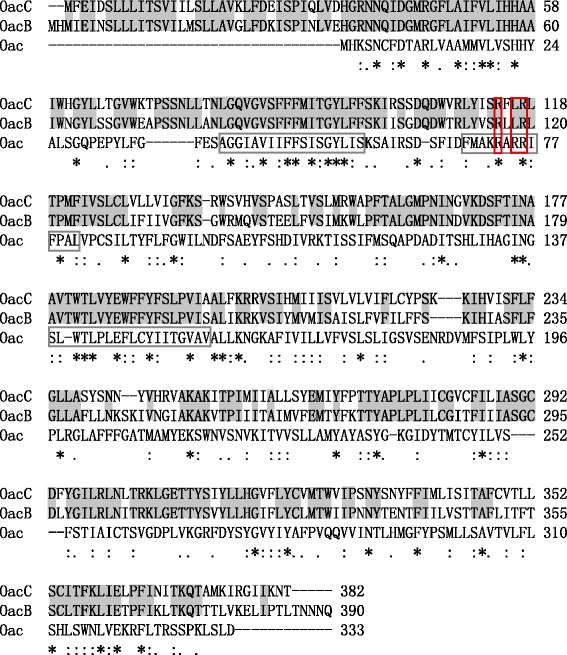
**Sequence alignment of OacB, OacC, and Oac (or OacA), the proteins involved in O-acetylation modification of** ***S. flexneri*** **.** Asterisks and dots indicate the amino acid residues that are identical or similar, respectively. Amino acids identical between OacB and OacC are shown in shadow. The three major regions conserved among the inner membrane trans-acylase family proteins are marked by black box. The three critical residues for the Oac (OacA) function are marked by red box.

Although the O-antigen of *S. flexneri* serotype 6 carries 3/4-O-acetylation on Rha^III^ [12,16,18], the *oacB* gene responsible for the same modification in serotypes 1a, 1b, 2a, 5a, and Y could not be detected from serotype 6 strains [16]. To identify potential factors associated with 3/4-O-acetylation of serotype 6, we used the OacB protein sequence of serotype 2a strain Sf301 (Accession No. NP_706267.1) to search against the GenBank protein database. A predicted acyltransferase protein encoded by gene SGF_00264 of *S. flexneri* strains CDC 796–83 and SFCCH060_3012 of CCH060 was found to show the highest homology (72% identity) to OacB (Figure 1). BLAST search revealed that these strains both carry within their genomes the serotype 6 O-antigen-specific gene *wzx* [21], indicating that they belong to serotype 6. Data presented below suggest that this OacB homolog is responsible for the 3/4-O-acetylation on Rha^III^ in serotype 6; hence it was named OacC and the encoding gene *oacC*, following the designations for OacB and *oacB* [16].

OacC possesses conserved domains of the acyltransferase family (COG1835 or acyl_trans_3). It showed 28-39% identity to predicted acyltransferases of *Pseudomonas* sp., *Dechloromonas aromatica*, *Flavobacterium columnare*, and some other species. OacC was also compared to Oac (which we have suggested to rename OacA [16]), an O-acetyltransferase responsible for 2-O-acetylation on Rha^I^ giving rise to group 6 antigenic determinant in *S. flexneri* serotypes 1b, 3a, 3b, 4b, and 7b [8,9]. The two proteins presented higher homology in the three regions conserved among the inner membrane *trans*-acylase family proteins (amino acid residues 40 to 57, 69 to 81, and 138 to 156 of Oac) [22] (Figure 1). In particular, the residues R73 and R76, which are known to be critical for Oac functioning [23], are conserved in the three known acyltransferases (Figure 1).

To reveal a correlation between the presence of *oacC* and 3/4-O-acetylation in serotype 6, we performed PCR screening using the *oacC-*1 primer pair of serotype 6 strains G1671, G1038, and 51579 (Table 1), all of which are known to carry 3/4-O-acetylation on Rha^III^. The expected PCR products (350-bp) were amplified from all three strains, and the PCR product sequences were fully identical to that of the SGF_00264 gene of CDC 796–83. PCR screening of another 74 serotype 6 isolates collected in our laboratory showed that all isolates were PCR positive (Table 2). The 74 strains were also positive for O-factor 9 (Table 2) and therefore should carry 3/4-O-acetylation on Rha^III^. These results, combined with the functional analysis data described below, suggest that OacC is responsible for the 3/4-O-acetylation on Rha^III^ in serotype 6.

**Table 1:** **Strains and plasmids used in this study**

**Strain or plasmid**	**Characteristic**	**Reference or source**
*S. flexneri*		
51579	Serotype 6, carrying 3/4-O-acetylation on Rha^III^, used for *oacC* gene cloning and inactivation analysis, Ap^s^, Km^s^	[19]
G1671, G1038	Serotype 6, carrying 3/4-O-acetylation on Rha^III^.	[18]
51579Δ*oacC*	Strain 51579 with the *oacC* gene replaced by the kanamycin resistance gene (*kan*) from pSR551, Km^r^, Ap^s^	this study
51579Δ*oacC*_pSQZ4	51579Δ*oacC* transformed by plasmid pSQZ4	this study
51579Δ*oacC*_pSQZ5	51579Δ*oacC* transformed by plasmid pSQZ5	this study
Sf301Δ*oacB*	Strain Sf301 with the *oacB* gene inactive, Km^r^, Ap^s^	[16]
Sf301Δ*oacB_*pSQZ4	Sf301Δ*oacB* transformed by plasmid pSQZ4	[16]
Sf301Δ*oacB_*pSQZ5	Sf301Δ*oacB* transformed by plasmid pSQZ5	this study
*E. coli*		
DH5α	*E. coli* strain used for plasmid propagation and gene cloning	TaKaRa
Plasmid		
pMD20T	T-A vector, Ap^r^	TaKaRa
pSR551	Km^r^, used for *kan* gene cloning	[24]
pKOBEG	A thermosensitive replicon that carries the λ phage *red*γβα operon expressed under the control of the arabinose-inducible pBAD promoter	[25]
pSQZ4	pMD20T carrying the whole sequence of the *oacB* gene from strain Sf301, Ap^r^	[16]
pSQZ5	pMD20T carrying the whole sequence of the *oacC* gene from strain 51579, Ap^r^	This study

**Table 2:** **PCR screening of** ***oacC*** **in various serotypes of** ***S. flexneri***

**Serotype**	**Number of strains tested**	**Number of O-factor 9 positive strains**	**Number of** ***oacB*** **PCR positive strains**	**Number of** ***oacC*** **PCR positive strains**
1a	106	102	102	0
1b	26	26	26	0
1c =7a	3	0	0	0
1d	14	0	0	0
2a	169	160	160	0
2b	61	0	0	0
3a	18	0	0	0
3b	4	0	0	0
4a	4	0	0	0
4av	4	0	0	0
4b	4	0	0	0
5a	14	9	9	0
5b	5	0	0	0
X	50	0	0	0
Xv	126	0	0	0
Y	39	24	24	0
Yv	20	0	0	0
6	77	77	0	77
7b	4	0	0	0

### Deletion and complementation analysis identifies the 3/4-O-acetylation function of *oacC* in serotype 6

To confirm the function of the *oacC* gene, we performed *oacC* deletion and complementation assay on serotype 6 strain 51579 using the one-step inactivation of chromosomal genes method [26]. The *kan-oacC* primer pair (Table 3) carrying sequences complementary to *oacC* was employed to amplify the aminoglycoside 3′-phosphotransferase encoding gene (Km^r^) from plasmid pRS551. The PCR amplicon (831-bp) transferred into strain 51579 would recombine with the *oacC* gene resulting in a part of the *oacC* gene sequence (599-bp, 217 to 815 base) being replaced by the Km^r^ gene. The deletion mutant 51579Δ*oacC* was selected on chloramphenicol and kanamycin-containing plate and detected by PCR amplification of the *oacC* gene using the *oacC*-1 and *oacC*-2 primer pairs (Table 3). The product of 350-bp which was amplified from the wild-type strain, was not obtained from the mutant 51579Δ*oacC* using the *oacC*-1 primer pair. However, when the *oacC*-2 primer pair was used, a PCR product of 1,681-bp was amplified from 51579Δ*oacC*, which, as expected, was longer than that from the wild-type strain 51579 (1,450-bp). The *oacC* deletion was further confirmed by sequencing analysis of the 1,681-bp amplicon from the 51579Δ*oacC* mutants.

**Table 3:** **Primers used in this study**

**Primer**	**Primer sequence (5′-3′)**	**Target gene**	**Reference**
*oacC*-1	F: gtgacacagtaagagaggc	*oacC*	NZ_AERO01000013
	R: tggaagaaataatcagatag		
*oacC*-2	F: ccgacgttccattagcccaaatctg	*oacC*	NZ_AERO01000013
	R: gcttccctgttcatagtggaacacc		
*kan-oacC*	F: gccatcttcgtacttattcatcatgccgctatttggcatggctacttattaaccggggtatggaaaactccgcacgttgtgtctcaaaatct	*kan*, *oacC*	NZ_AERO01000013
	R: cctgatgcgataagtataaagcaaacaccgcaaattatgagagggagtggagcgtagcgtcccgtcaagtcagcgta		
*oacC-*3	F: cccctgcctctcttactgtg	*oacC,*SGF_00268	NZ_AERO01000013
	R: gaatatgctgcctgacctgt		
*oacC-*4	F: cagtaagagaggcaggggag	*oacC*, SFCCH060_3017	NZ_AKMW01000058
	R: gggcataagcagggcaagag		

The serological features of the mutants were determined by an agglutination assay using *Shigella* antisera of Seiken and anti-O-factor 9 serum. The deletion of *oacC* did not affect the type VI antigenicity, with the 51579Δ*oacC* mutant presenting the same antiserum VI reactivity as the parental 51579 (Table 4). This observation is consistent with and confirm the results of Hygge Blakeman *et al.* [27], who found that *E. coli* O147 (which possesses the same structure as that of serotype 6 O-antigen except that lacking O-acetylation on Rha^III^) present the same monoclonal antibody MASF VI-1 reactivity. In contrast, compared to the wild type, the mutant lost the reactivity with 3/4-O-acetylated Rha^III^-specific anti-O-factor 9 serum (Table 4). The O-factor 9 antigenicity of the 51579Δ*oacC* mutant was restored by complementation with a functional *oacC*-carrying plasmid pSQZ5 (Table 4).

**Table 4:** **Serotyping of wild-type strains,** ***oacC*** **and** ***oacB*** **deletion mutants, and complementation transformants by plasmid pSQZ4 or pSQZ5**

**Strains**	**Reactivity with typing and grouping antisera**									
	**I**	**II**	**III**	**IV**	**V**	**VI**	**3,4**	**6**	**7,8**	**9**
51579	-	-	-	-	-	+	-	-	-	+
51579Δ*oacC*	-	-	-	-	-	+	-	-	-	-
51579Δ*oacC_* pSQZ4	-	-	-	-	-	+	-	-	-	+
51579Δ*oacC_* pSQZ5	-	-	-	-	-	+	-	-	-	+
Sf301	-	+	-	-	-	-	+	-	-	+
Sf301Δ*oacB* [16]	-	+	-	-	-	-	+	-	-	-
Sf301Δ*oacB*_pSQZ4 [16]	-	+	-	-	-	-	+	-	-	+
Sf301Δ*oacB*_pSQZ5	-	+	-	-	-	-	+	-	-	+

**Figure 2:**
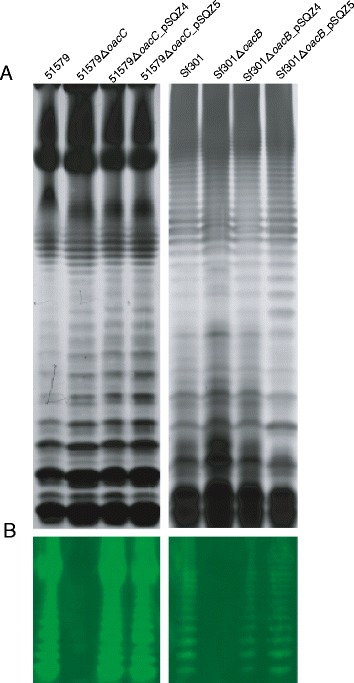
**SDS-PAGE and Western blot of LPS from wild-type strains 51579 (serotype 6) and Sf301 (serotype 2a), their** ***oacC*** **and** ***oacB*** **deletion mutants, and complementation transformants by plasmids pSQZ4 and pSQZ5. A**. Sliver-staining detection of LPS profiles on 15% polyacrylamide gels. **B**. The LPS separated by SDS-PAGE were transferred onto a PVDF membrane and hybridized with anti-O-factor 9 serum. An anti-rabbit antibody labeled with fluorescent IRDye™ 800 (Rockland) was used as the secondary antibody. Fluorescence was detected using Odyssey Infrared Imaging System (LI-COR).

The O-factor 9 antigenicity was further confirmed by an immunoblotting assay. The LPSs samples were resolved on 15% SDS-PAGE gel and visualized by silver-staining (Figure 2A). A typical ladder-like banding pattern of an LPS with an O-antigen composed of various numbers of O-units was observed for all strains with no obvious difference between the parental strain and *oacC* deletion and complementation mutants. In Western blot, anti-O-factor 9 serum recognized the ladder-like LPS bands of the functional *oacC*-carrying strains (51579 wild type and 51579Δ*oacC_*pSQZ5 complementation mutant) but not the 51579Δ*oacC* deletion mutant (Figure 2B).

**Figure 3:**
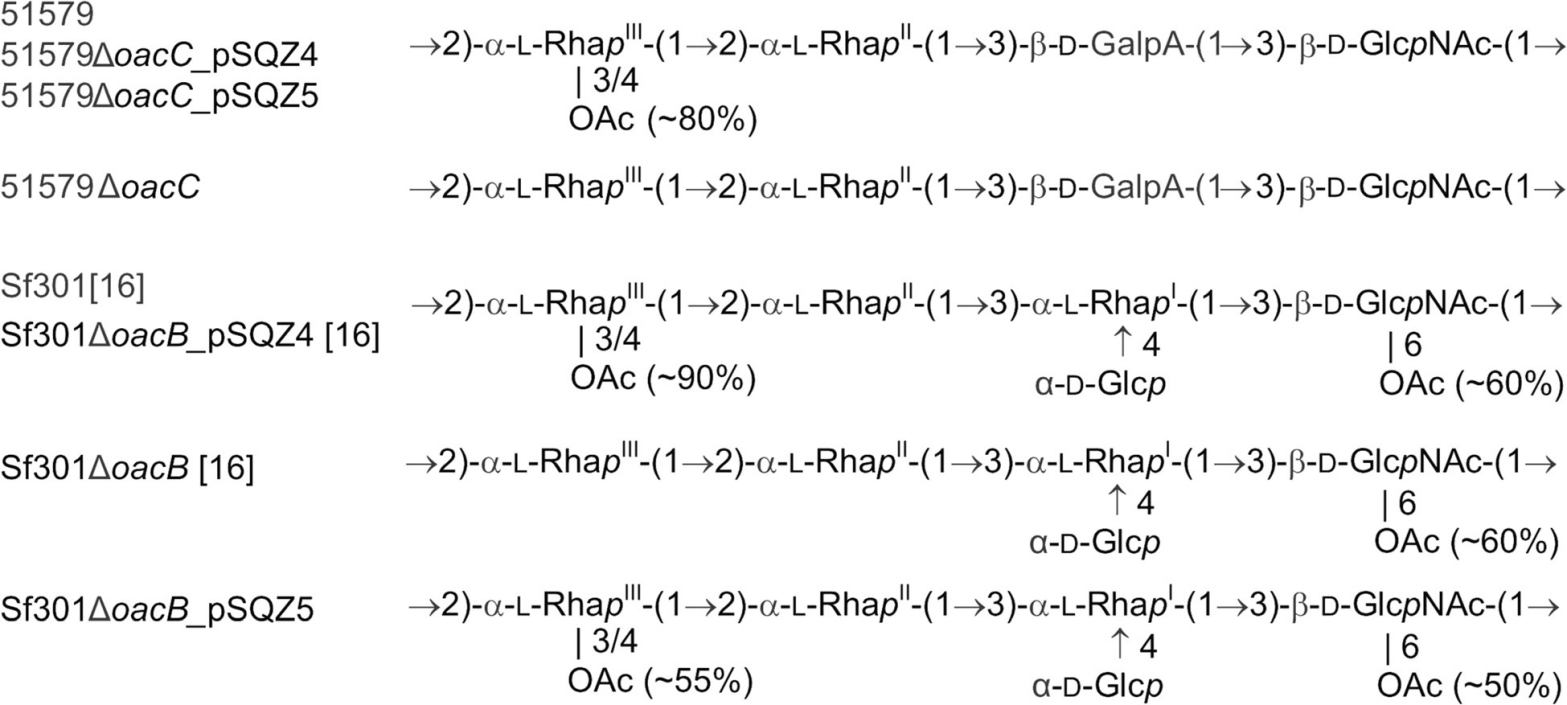
**O-Polysaccharide structures of wild-type strains 51579 (serotype 6) and Sf301 (serotype 2a), their** ***oacC*** **and** ***oacB*** **deletion mutants, and complementation transformants by plasmids pSQZ4 and pSQZ5** [16]**.**

These findings were confirmed by structure analysis using ^1^H and ^13^C NMR spectroscopy of the O-polysaccharides isolated from the LPSs. In the spectra of the wild-type 51579 O-polysaccharide, there were signals for the 3-O-acetyl group (major) and 4-O-acetyl group (minor) at δ_H_ 2.14 and 2.19, δ_С_ 21.9 and 21.8, respectively. Due to electron deshielding effects of O-acetylation [28], parts of the signals for H-3/C-3 and H-4/C-4 of Rha^III^ were shifted downfield to δ_H_/δ_С_ 5.04/74.2 and 4.80/75.7 as compared with their positions in the non-O-acetylated Rha^III^ at δ_H_/δ_С_ 3.83/71.2 and 3.38/73.6, respectively (compare published data for serotype 6a strain G1671 [18]). The degrees of O-acetylation determined by relative intensities of the ^1^H NMR signals for various O-acetylated and non-O-acetylated Rha^III^ forms were ~50% at position 3 and ~30% at position 4. The ^1^H and ^13^C NMR spectra of the 51579Δ*oacC* mutant O-polysaccharide lacked any signals for O-acetyl groups, and the positions of resonances of H-3/C-3 and H-4/C-4 of Rha^III^ at δ_H_/δ_С_ 3.84/71.2 and 3.36/73.7, respectively, indicated that they did not undergo any deshielding. The spectra of the 51579Δ*oacC_*pSQZ5 transformant were essentially identical to those of the wild type. Therefore, the 51579Δ*oacC* mutant lost the 3/4-O-acetylation on Rha^III^, and this modification was restored by complementation of the mutant with a functional *oacC* gene (Figure 3).

It has been proposed that the degree of 3/4-O-acetylation on Rha^III^ contributes to the serospecificity of serotype 6, which can be divided into subtypes 6 and 6a with low and high degree of 3/4-O-acetylation, respectively [18]. We amplified and sequenced the *oacC* gene in serotype 6a strain G1671 and serotype 6 strain G1038 [18], and found no nucleotide difference between them. Hence, the variation in the degree of 3/4-O-acetylation is not due to sequence variation of the *oacC* gene, and most likely, results from different storage and/or cultivation conditions.

### *oacC* of serotype 6 has the interchangeable 3/4-O-acetylation function with *oacB* of serotypes 1a, 1b, 2a, 5a, and Y

To determine the distribution of *oacC* in *S. flexneri*, PCR screening of 671 strains of various non-6 serotypes (Table 2) was performed using the *oacC-*1 primer pair. No positive amplification was observed in any of the strains tested, including all 321 O-factor 9 positive strains of serotypes 1a, 1b, 2a, 5a, and Y, which do carry 3/4-O-acetylation (Table 2). We also PCR screened 12 strains of *S. dysenteriae* (one each of serotypes 1 to 12), 18 strains of *S. boydii* (one each of serotypes 1 to 18), 31 strains of *S. sonnei* and 10 strains of *E. coli* (one each of serogroups O6, O8, O13, O44, O71, O78, O127, O128, O157, O159) (Additional file 1: Table S1), and found that all were *oacC* negative. These data, combined with results of our previous studies on the *oacB* distribution [20], indicate that *oacC* is specific to serotype 6, whereas *oacB* to the other serotypes of *S. flexneri*.

To elucidate whether the OacC protein has the interchangeable 3/4-O-acetylation function with OacB, we transferred the *oacC*-carrying plasmid pSQZ5 into Sf301Δ*oacB* (serotype 2a strain Sf301 whose *oacB* gene is inactivated) [16], and the *oacB*-carrying plasmid pSQZ4 [16] into 51579Δ*oacC*, to construct complementation transformants Sf301Δ*oacB_* pSQZ5 and 51579Δ*oacC_* pSQZ4, respectively (Table 1)*.* The serological features of the transformants were determined by an agglutination assay using *Shigella* antisera of Seiken (Danka Seiken, Japan) and anti-O-factor 9 serum, and compared to those of the Sf301Δ*oacB_* pSQZ4 [16] and 51579Δ*oacC_* pSQZ5 transformants (see above). It was found that both Sf301Δ*oacB_* pSQZ5 and 51579Δ*oacC_* pSQZ4 acquired the agglutination reactivity with anti-O-factor 9 serum, and the serological features of the *oacC* transformants Sf301Δ*oacB_* pSQZ5 and 51579Δ*oacC_* pSQZ5 were same as those of the *oacB* transformants Sf301Δ*oacB_* pSQZ4 and 51579Δ*oacC_* pSQZ4, respectively (Table 4). In the immunoblotting assay, anti-O-factor 9 serum recognized the ladder-like LPS bands of Sf301Δ*oacB_* pSQZ4, Sf301Δ*oacB_* pSQZ5, 51579Δ*oacC_* pSQZ4, and 51579Δ*oacC_* pSQZ5 with no obvious difference found between the *oacB* and *oacC* transformants in each host (Figure 2B).

The ability of *oacB* and *oacC* to transform the deletion mutants of the heterologous serotypes was further confirmed by ^1^H and ^13^C NMR spectroscopy, which showed that the O-polysaccharides of the transformants had acquired the 3/4-O-acetylation on Rha^III^ (Figure 3). The ^1^H and ^13^C NMR spectra of the O-polysaccharides of 51579Δ*oacC_* pSQZ5 (characterized above in mutation and complementation analysis) and 51579Δ*oacC_* pSQZ4 were essentially identical, and, hence, the O-polysaccharides had the same structure, including the same total degree of 3/4-O-acetylation (~80% in both). Serotype 2a strain Sf301, its Sf301Δ*oacB* deletion mutant, and the Sf301Δ*oacB_* pSQZ4 transformant has been characterized by us earlier, and the ^1^H and ^13^C NMR chemical shifts of their O-polysaccharides have been reported [16]. The ^1^H and ^13^C NMR spectra of the O-polysaccharide of Sf301Δ*oacB_* pSQZ5 were similar to those of Sf301Δ*oacB_* pSQZ4, differing only in lower intensities in the former strain of the signals for the 3-O-acetyl and 4-O-acetyl groups at δ_H_ 2.20 and 2.14, δ_С_ 22.0 and 22.1, as well as for the O-acetylated forms of Rha^III^: H-3/C-3 at δ_H_/δ_С_ 5.09/74.1 and H-4/C-4 at δ_H_/δ_С_ 4.80/75.8. A lower degree of O-acetylation in the Sf301Δ*oacB* mutant complemented with the heterologous *oacC* gene as compared with the homologous *oacB* gene (totally ~55% versus ~90% [16]) could be due to a poorer adaptation of OacC of serotype 6 for 3/4-O-acetylation of the serotype 2a O-polysaccharide having a different backbone structure.

### Gene *oacC* is carried by a bacteriophage structure on the chromosome

**Figure 4:**
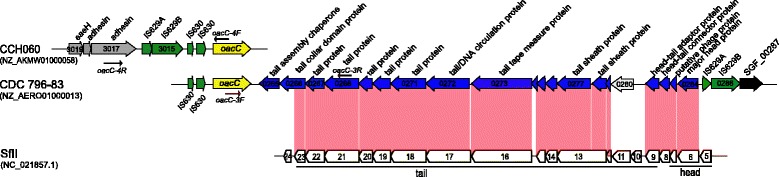
**Genetic structures of the genomic regions franking** ***oacC*** **gene in serotype 6 strains.** Sequences of contig NZ_AERO01000013 (strain CDC796-83), NZ_AKMW01000058 (CCH060), and SfII (NC_021857.1) were obtained from NCBI database. The *orfs* were annotated as submitted sequences in NCBI, and shown as thick arrows. The conserved genes of NZ_AERO01000013 and NZ_AKMW01000058 were shown in different colors: phage original genes, blue; IS629 and IS630, green; *oacC*, yellow; pseudo, gray; others, black. Genes sharing >95% identity at amino acid level between CDC796-83 and serotype-converting bacteriophage SfII are marked by red shadow. Functional domains of SfII are indicated below. Key primers used for PCR screening are indicated by thin arrows. The locus_tag numbers are showed in the arrows, and the encoded proteins are indicated above.

The DNA regions flanking the *oacC* gene of partially sequenced serotype 6 strains CDC 796–83 and CCH060 were analyzed, and the genomic structures are shown in Figure 4. In strain CDC 796–83, the *oacC* gene was located at the 5′ end of contig NZ_AERO01000013 and immediately followed by 19 *orfs* encoding proteins of phage origin, with 15 of tail structure and assembly (SGF_00265- SGF_00279), two corresponding to head-tail adaptor/connector (SGF_00281, SGF_00282), one putative phage protein (SGF_00283), and one related to head assembly (SGF_00284) (Figure 4, Additional file 2: Table S2). This prophage structure is homologous to (>95% identity at protein level), and organized in a similar manner as, the structure region (SfII_6-SfII_23) of *S. flexneri* bacteriophage SfII genome (NC_021857.1) (Figure 4), indicating their close evolutionary relationship. In contrast, it showed no similarity to the *oac*-carrying bacteriophage Sf6 and the *oac1b*-carrying phage-like structure, which are both responsible for the 2-O-acetylation on Rha^I^ in serotypes 1b, 3a, 3b, 4b, and 7b [17,29], or to the transposon-like structure carrying *oacB* for 3/4-O-acetylation of Rha^III^ in serotypes 1a, 1b, 2a, 5a, and Y [16].

To elucidate whether the *oacC*-carrying prophage structure occurs in all serotype 6 strains, we performed PCR screening on 77 serotype 6 strains using the *oacC-*3 primer pair (Table 3), which covers the *oacC* gene and neighboring SGF_00268 genes. Except for 12 strains, the expected product (3,850-bp) could be amplified from all strains tested, and the PCR product sequences were identical to that of contig NZ_AERO01000013. The *oacC* gene of strain CCH060 was found to be located near the end of contig NZ_AKMW01000058 downstream of two insertion sequences (IS629 and IS630). They followed a region of genes encoding putative adhesin and attaching/effacing protein (SFCCH060_3018- SFCCH060_3019) (Figure 4), a fragment of unknown function that was also present in the genomes of several *E. coli* strains (ES11, LY180, W, and KO11). The same insertion sequence IS630 also occurred immediately upstream of *oacC* in strain CDC 796–83 (Figure 4). Furthermore, PCR amplification and sequencing performed on the 77 serotype 6 strains using the *oacC-*4 primer pair that covers SFCCH060_3012 (*oacC*) and SFCCH060_3017, showed that all but 4 strains (HN157, HN12, G1038 and 51579) tested were PCR positive. The four outliers also were negative when further PCR amplification was performed targeting on genes up to SFCCH060_3019, probably owing to DNA fragment insertion or deletion events happened in this region. PCR screening using the *oacC-*3 primer pair indicated that the four strains carried the phage-like structure downstream of *oacC*, and, therefore, might acquire the 3/4-O-acetylation by the same phage mechanism.

## Conclusions

In this work, the *oacC* gene encoding a novel acyltransferase OacC was demonstrated to be responsible for the 3/4-O-acetylation on Rha^III^ in *S. flexneri* serotype 6. This conclusion was supported by the following evidences: i) the OacC protein encoded by the *oacC* gene showed significant similarity to OacB and other acyltransferase family proteins; ii) deletion of the functional *oacC* gene resulted in the loss of the 3/4-O-acetylation in serotype 6; iii) the cloned *oacC* gene mediated the 3/4-O-acetylation of Rha^III^ upon transformation. The *oacC* gene is specific to serotype 6 and presented in all strains of this serotype tested; hence, this gene can be used as a target for molecular identification of *S. flexneri* serotype 6.

Although *oacC* occurs only in serotype 6 strains, it has the interchangeable function with *oacB*, which is responsible for 3/4-O-acetylation of Rha^III^ in non-6 *S. flexneri* serotypes, and conversely *oacB* can confer this modification to serotype 6. This could be expected as *oacC* and *oacB* possess a high homology (72% identity), and the O-polysaccharides of serotype 6 and the other *S. flexneri* serotypes share the → 2)-α-l-Rha*p*^III^-(1 → 2)-α-l-Rha*p*^II^ disaccharide fragment.

As opposite to the *oacB* gene localized in a transposon-like structure, the *oacC* gene is carried by a chromosomal phage-like structure adjacent to the adhesin region that is conserved in serotype 6. Therefore, the mechanisms of 3/4-O-acetylation in serotype 6 and the other serotypes (1, 2, 5 and Y) are distinct, which is not surprising as they have different evolutionary origins and belong to different lineages of *Shigella* clones of *E. coli* [13,14]. Accordingly, the divergent Oacs might have been gained from different species in independent events. These findings enhance our understanding of the genetic basis of O-antigen modifications in *S. flexneri.*

## Methods

### Bacterial strains, plasmids, and culturing conditions

Strains and plasmids used in this study are listed in Table 1. *S. flexneri* serotype 6 strain 51579 [16] carrying 3/4-O-acetylation on Rha^III^ in the O-antigen was used as the reference strain for *oacC* gene cloning and deletion analysis. The *oacC* gene deletion mutant 51579Δ*oacC* and *oacB* gene deletion mutant Sf301Δ*oacB* [16] were employed as hosts for the plasmid pSQZ4 and pSQZ5 transformation analysis. Seventy-seven *S. flexneri* serotype 6 strains, 671 strains of other serotypes of *S. flexneri* (Table 2), 12 strains of *S. dysenteriae* (one each of serotypes 1 to 12), 18 strains of *S. boydii* (one each of serotypes 1 to 18), 31 strains of *S. sonnei* and 10 strains of *E. coli* (one each of serogroups O6, O8, O13, O42, O71, O78, O127, O128, O157, O159) were used for *oacC* gene PCR detection analysis (Additional file 1: Table S1). *S. flexneri* strains were either isolates from patients in a surveillance program performed by China CDC during 2000 to 2012 or were purchased from the National Collection of Type Cultures (NCTC) or were kindly donated by B. Liu (Nankai University, Tianjin). *E. coli* DH5α was used for plasmid propagation. pMD20T vector (TaKaRa, Japan) was used for DNA sequencing and *oacC* gene function analysis. Plasmid pRS551 was used for kanamycin resistance gene amplification. pKOBEG encoding a homologous recombination system was used in *oacC* gene deletion analysis. Strains were grown in a 37°C incubator or orbital shaker in Luria-Bertani (LB) medium supplemented with ampicillin (100 μg ml^−1^), kanamycin (40 μg ml^−1^), or chloramphenicol (50 μg ml^−1^) when appropriate.

### Bioinformatics analysis

The protein sequence of O-acyltransferase for 3/4-O-acetylation (OacB) (accession No. NP_706267.1) of *S. flexneri* strain Sf301 (serotype 2a) was searched against the GenBank protein database, using the BLASTP web server (http://www.ncbi.nlm.nih.gov/BLAST). Homologs of OacB were aligned using the ClustalW2 program in the EMBL-EBI (http://www.ebi.ac.uk/Tools/msa/clustalw2/).

### DNA techniques

Primer pairs used in this study are listed in Table 3. The *oacC*-1 primer pair was used for *oacC* gene detection. The *oacC*-2 primer pair was used for *oacC* gene function analysis. The *oacC-*3 and *oacC-*4 primer pairs were used to amplify regions up and downstream of *oacC* in serotype 6 isolates. Oligonucleotide primers were synthesized by Sangon Biotech (Shanghai). PCR amplifications were performed using a TaKaRa PCR Amplification Kit (Takara, Japan) following a standard protocol. PCR products amplified from strain 51579 using the *oacC*-2 primer pair were purified and cloned into the T-vector pMD20T (TaKaRa, Japan), which carries an additional T at both 3′ terminus and can complement the A base of the PCR product, to generate the pSQZ5 expression plasmid. The recombinant plasmids were first transformed into commercial *E. coli* DH5α competent cells (TaKaRa, Japan), and then into *S. flexneri* strains tested, using a standard protocol [30]. The transformants were selected on LB plates supplemented with ampicillin (100 μg ml^−1^) and further confirmed by PCR amplification of the *oacC* gene.

### *oacC* gene functional deletion and complementation analysis

Deletion of the *oacC* gene was performed on *S. flexneri* serotype 6 strain 51579 using a one-step method as described previously [26]. The kanamycin resistance gene (Km^r^) was PCR amplified from plasmid pRS551 using the *kan-oacC* primer pair (Table 3). The PCR products were electroporated into strain 51579 carrying plasmid pKOBEG (encoding a homologous recombination system for *oacC* gene inactivation) and selected on an LB plate with chloramphenicol (50 μg ml^−1^) and kanamycin (40 μg ml^−1^). *oacC* gene deletion mutant 51579Δ*oacC* was confirmed by a slide agglutination assay using anti-O-factor 9 serum [20] and PCR amplification of *oacC* using the *oacC*-1 and *oacC*-2 primer pairs. Plasmid pSQZ5 and pSQZ4 [16] were transferred into 51579Δ*oacC* and Sf301Δ*oacB* [16], giving rise to complemented strains 51579Δ*oacC*_ pSQZ5, 51579Δ*oacC*_ pSQZ4 and Sf301Δ*oacB*_pSQZ5, respectively.

### Serotyping analysis

The serological features of *S. flexneri* strains were determined by a slide agglutination test using commercially available *Shigella* monovalent antisera kit (Denka Seiken, Japan) and 3/4-O-acetylated Rha^III^-specific anti-O-factor 9 serum prepared previously [20].

### Western blot assay

LPSs were prepared using an LPS extraction Kit (iNtRON, South Korea) according to the manufacturer’s instructions. The LPSs were electrophoresed on 15% polyacrylamide gels and detected by silver staining as described [31]. A Western blot assay of the LPSs was performed as described [20]. Briefly, the LPSs separated by SDS-PAGE were transferred onto a polyvinylidene difluoride (PVDF) and incubated with anti-O-factor 9 serum. After washing, the membrane was incubated with anti-rabbit antibody labeled with fluorescent IRDye 800 (Rockland). The fluorescence was detected using an Odyssey infrared imaging system (LI-COR).

### O-polysaccharide isolation and structure analysis

For O-polysaccharide structure analysis, the LPSs of wild-type strains, deletion mutants and transformants were isolated by phenol-water extraction of bacterial cells [32]. The crude extract without separation of layers was dialyzed against tap water, nucleic acids and proteins were precipitated by adding aqueous 50% CCl_3_CO_2_H at 4°C to reach pH 2, the supernatant was dialyzed against distilled water and freeze-dried. The purified LPSs obtained in yields of 5% to 7% were hydrolyzed with aqueous 2% acetic acid at 100°C until formation of a lipid precipitate (1–2 h), and the released O-polysaccharides were isolated in yields of 12% to 34% from the supernatant by gel permeation chromatography on Sephadex G-50 Superfine (Amersham Biosciences, Sweden) in 0.05 M pyridinium acetate buffer (pH 4.5) monitored with a differential refractometer (Knauer, Germany).

Structures of the O-polysaccharides were elucidated using two-dimensional nuclear magnetic resonance (NMR) spectroscopy, including a ^1^H,^13^C heteronuclear single-quantum coherence (HSQC) experiment, essentially as described previously [33]. Positions of O-acetyl groups were determined by characteristic low-field displacements of NMR signals for ^1^H and ^13^C atoms at the O-acetylation sites. The degree of O-acetylation was determined by relative integral intensities of the ^1^H NMR signals for the O- and N-acetyl groups, 3-O- and 4-O-acetylated and non-O-acetylated Rha^III^.

### Availability of supporting data

All supporting data are available and included as additional files (Additional file 1: Table S1 and Additional file 2: Table S2).

## Additional files

Additional file 1: Table S1.
*Shigella* and *E. coli* strains used for the *oacC* gene PCR detection and anti-O-factor 9 serum specificity evaluation. Additional file 2: Table S2. Analysis of predicted ORFs of *oacC*-carrying contig NZ_AERO01000013 of serotype 6 strain CDC 796–83.
