# Supplementary material for: Genetic and structural identification of an O-acyltransferase gene (oacC) responsible for the 3/4-O-acetylation on rhamnose III in Shigella flexneri serotype 6
Source: BMC Microbiol. 2014 Oct 21;14:266. doi: 10.1186/s12866-014-0266-7 (PMC4206707; doi:10.1186/s12866-014-0266-7)
Supplement: Additional file 1: Table S1. — Shigella and E. coli strains used for the oacC gene PCR detection and anti-O-factor 9 serum specificity evaluation. [file 12866_2014_266_MOESM1_ESM.pdf]

Supplementary Table 1 *Shigella* and *E. coli* strains used for the *oacC* gene PCR detection and anti-O-factor 9 serum specificity evaluation

Strains[a]	Species	Serotypes	*oacC* gene PCR results	Reactivity with antiserum 9
51197	*S. dysenteriae*	A1	-	-
51136	*S. dysenteriae*	A2	-	-
51196	*S. dysenteriae*	A3	-	-
51253	*S. dysenteriae*	A4	-	-
51252	*S. dysenteriae*	A5	-	-
51258	*S. dysenteriae*	A6	-	-
51259	*S. dysenteriae*	A7	-	-
51526	*S. dysenteriae*	A8	-	-
51527	*S. dysenteriae*	A9	-	-
51528	*S. dysenteriae*	A10	-	-
51630	*S. dysenteriae*	A11	-	-
51631	*S. dysenteriae*	A12	-	-
51360	*S. boydii*	C1	-	-
51361	*S. boydii*	C2	-	-
51226	*S. boydii*	C3	-	-
3594-74	*S. boydii*	C4	-	-
51227	*S. boydii*	C5	-	-
51228	*S. boydii*	C6	-	-
51586	*S. boydii*	C7	-	-
51587	*S. boydii*	C8	-	-
51588	*S. boydii*	C9	-	-
51469	*S. boydii*	C10	-	-
51589	*S. boydii*	C11	-	-
51590	*S. boydii*	C12	-	-
51531	*S. boydii*	C13	-	-
51532	*S. boydii*	C14	-	-
51591	*S. boydii*	C15	-	-
51632	*S. boydii*	C16	-	-
51633	*S. boydii*	C17	-	-
51634	*S. boydii*	C18	-	-

51081	*S. sonnei*	I	-	-
03HL14	*S. sonnei*	I	-	-
1978GZ02	*S. sonnei*	I	-	-
2005163	*S. sonnei*	II	-	-
2005186	*S. sonnei*	II	-	-
09GS057	*S. sonnei*	II	-	-
09GS058	*S. sonnei*	II	-	-
09GS076	*S. sonnei*	II	-	-
09GS078	*S. sonnei*	II	-	-
1978GZ04	*S. sonnei*	II	-	-
1982GZ05	*S. sonnei*	II	-	-
2008GZ04	*S. sonnei*	II	-	-
2009GS081	*S. sonnei*	II	-	-
2009GS083	*S. sonnei*	II	-	-
2009GS086	*S. sonnei*	II	-	-
2009GS088	*S. sonnei*	II	-	-
2009GS090	*S. sonnei*	II	-	-
2009GS094	*S. sonnei*	II	-	-
2009GS095	*S. sonnei*	II	-	-
2009GS098	*S. sonnei*	II	-	-
2009GS100	*S. sonnei*	II	-	-
2009GZ16	*S. sonnei*	II	-	-
2009GZ20	*S. sonnei*	II	-	-
2009GZ26	*S. sonnei*	II	-	-
2009GZ31	*S. sonnei*	II	-	-
2009GZ46	*S. sonnei*	II	-	-
2009GZ47	*S. sonnei*	II	-	-
2010GZ24	*S. sonnei*	II	-	-
2010GZ26	*S. sonnei*	II	-	-
2010GZ27	*S. sonnei*	II	-	-
M6381	*S. sonnei*	II	-	-
ET57	*E. coli*	O6	-	-
ET06	*E. coli*	O8	-	-

G1237	*E. coli*	O13	-	-
042	Enteroaggregative *E. coli*	O44:H18	-	-
ET03	*E. coli*	O71	-	-
MH1278-15	*E. coli*	O78	-	-
EP001	*E. coli*	O127	-	-
ET47	*E. coli*	O128	-	-
EDL933	Enterohemorrhagic *E. coli*	O157:H7	-	-
ET100	*E. coli*	O159	-	-

[a], all the strains were isolated from diarrhea patients in China, or purchased from the National Institutes for Food and Drug Control (NIFDC).
