# Supplementary material for: Genetic and structural identification of an O-acyltransferase gene (oacC) responsible for the 3/4-O-acetylation on rhamnose III in Shigella flexneri serotype 6
Source: BMC Microbiol. 2014 Oct 21;14:266. doi: 10.1186/s12866-014-0266-7 (PMC4206707; doi:10.1186/s12866-014-0266-7)
Supplement: Additional file 2: Table S2. — Analysis of predicted ORFs of oacC-carrying contig NZ_AERO01000013 of serotype 6 strain CDC 796–83. [file 12866_2014_266_MOESM2_ESM.pdf]

Supplementary Table 2 Alignment analysis of genomic region franking *oacC* gene in serotype 6 strain CDC 796-83

ORFs[a]	No. amino acid encoded	Database search results	
		Protein or origin (species, accession no.)	Identity (%)
0262	52	integrease protein (*S. enterica*, WP_001853537)	92
0263	87	transposase (*S. flexneri*, WP_000774867.1)	99
0264	382	OacB (*S. flexneri* 2a str. 301, NP_706267)	72
0265	203	tail assembly chaperone gp38 (*E. coli* ATCC 8739, YP_001725714)	96
0266	260	phage tail collar domain protein (*E. coli*, WP_001494081)	99
		SfII_23, (SfII, AGQ45513)	94
0267	194	tail protein (SfV, NP_599052)	99
		tail protein (SfII, YP_008318500)	98
0268	352	tail protein (SfV, NP_599051)	98
		baseplate protein (SfIV, YP_008766882)	97
0269	141	tail protein (SfV, NP_599050)	99
		tail protein (SfII, YP_008318498)	98
0270	182	tail protein (SfV, NP_599049)	100
0271	359	putative phage tail protein (*E. coli*, YP_007379831)	99
0272	442	phage tail/DNA circulation protein (*E. coli*, WP_021528953)	99
0273	611	tail protein (SfV, NP_599046)	99
		phage tail tape measure protein (*E. coli*, WP_001583206)	99
0274	62	hypothetical protein (*E. coli*, WP_001303047)	98
0275	89	hypothetical protein SfVp13 (SfV, NP_599045)	99
0276	118	hypothetical protein SfVp12 (SfV, NP_599044)	98

0277	337	tail sheath protein (*E. coli*, WP_001472772)	99
0278	142	tail sheath protein (*E. coli*, WP_000155729)	100
0279	46	hypothetical protein (*E. coli*, WP_001542465)	98
		hypothetical protein SfVp10 (SfV, NP_599042)	98
0280		pseudo	
0281	136	head tail adaptor protein (SfII, YP_008318487)	95
0282	107	head-tail connector protein (SfII, YP_008318486)	94
0283	66	Putative phage protein SfII_007 (SfII, AFR52515)	97
0284	203	major head protein (SfIV, YP_008766869)	99
0285	91	transposase (*E. coli*, WP_001370105)	100
0286	295	IS629 ORF2 (*S. boydii*, YP_406278)	100
0287	227	diacetylchitobiose permease (*E. coli*, WP_000379769)	99
		PTS N'-diacetylchitobiose transporter subunit IIC (*E. coli*, WP_001338139)	99

[a], ORFs are showed as locus_tag numbers annotated in contig NZ_AERO01000013 of strain CDC796-83
